# The Effect of Partial Electrical Insulation of the Tip and Active Needle Length of Monopolar Irreversible Electroporation Electrodes on the Electric Field Line Pattern and Temperature Gradient to Improve Treatment Control

**DOI:** 10.3390/cancers15174280

**Published:** 2023-08-26

**Authors:** Annemiek M. Hogenes, Cornelis H. Slump, Gerben A. te Riet o. g. Scholten, Martijn W. J. Stommel, Jurgen J. Fütterer, Rudolf M. Verdaasdonk

**Affiliations:** 1 Department of Medical Imaging, Radboud University Medical Center, P.O. Box 9101 (766), 6500 HB Nijmegen, The Netherlands; 2 Department of Robotics and Mechatronics, University of Twente, 7522 NB Enschede, The Netherlands; 3 Department of Surgery, Radboud University Medical Center, 6525 GA Nijmegen, The Netherlands; 4 Department of Health Technology Implementation, TechMed Center, University of Twente, 7522 NB Enschede, The Netherlands

**Keywords:** irreversible electroporation, electrode, electrical insulation, thermal effects, temperature gradient, electric field line pattern, phantom model

## Abstract

**Simple Summary:**

To control and reduce the impact of thermal effects on critical structures in the vicinity of irreversible electroporation (IRE) electrodes, the effect of partial electrical insulation of the original monopolar IRE electrodes (tip and/or part of the active needle length (ANL)) was investigated and visualized. Electric field lines strongly fanned out and showed the highest density near the electrode tip. Without any additional insulation, the highest change in temperature gradient was visualized near the electrode tip, indicating a risk for thermal effects. A combination of ANL and tip insulation reduced the area where current and heat release can occur, leading to an increase in temperature (gradient) at the uninsulated ANL. Electrically insulating the electrode tip could offer an improvement in treatment control, predictability where current pathways are formed. Further research on the tip insulation method and clinical applicability will prove this innovation’s future.

**Abstract:**

Unintentional local temperature effects can occur during irreversible electroporation (IRE) treatment, especially near the electrodes, and most frequently near the tip. Partial electrical insulation of the IRE electrodes could possibly control these temperature effects. This study investigated and visualized the effect of partial electrical insulation applied to the IRE electrodes on the electric field line pattern and temperature gradient. Six designs of (partial) electrical insulation of the electrode tip and/or active needle length (ANL) of the original monopolar 19G IRE electrodes were investigated. A semolina in castor oil model was used to visualize the electric field line pattern in a high-voltage static electric field. An optical method to visualize a change in temperature gradient (color Schlieren) was used to image the temperature development in a polyacrylamide gel. Computational models were used to support the experimental findings. Around the electrode tip, the highest electric field line density and temperature gradient were present. The more insulation was applied to the electrodes, the higher the resistance. Tip and ANL insulation together reduced the active area of and around the electrodes, resulting in a visually enlarged area that showed a change in temperature gradient. Electrically insulating the electrode tip together with an adjustment in IRE parameter settings could potentially reduce the uncontrollable influence of the tip and may improve the predictability of the current pathway development.

## 1. Introduction

The efficacy of IRE relies on tumor cell death through permanent permeabilization of cell membranes by high-voltage electrical pulses that change the transmembrane potential [1,2]. Inducing irreversible electroporation (IRE), where the applied voltage is as high as possible, providing a safe treatment without collateral damage due to thermal effects, is a delicate balance. Given the mode of action, IRE has the potential to ablate tumors near critical structures, for example, in the liver hilum or near the central bile ducts [3]. These critical structures are not damaged since the extracellular matrix remains intact [4,5,6]. Although the initial effect of IRE does not rely on a temperature increase, a temperature rise does occur in practice [7,8,9]. The threshold between IRE and thermal ablation is a grey area [10], dependent on the applied electric field strength [11], pulse parameters (e.g., pulse duration and electric field exposure duration [11]), physical properties of the tumor tissue (e.g., blood perfusion and electrical conductivity [12,13]) as well as the amount of energy in joules that has been delivered to the tissue [14]. The electric field is the strongest and shows the highest electric field line density near the electrodes [15,16]. This is the reason why temperature effects occur near the electrodes [17]. To prevent collateral damage, it is advised to place the needle electrodes 2 mm from vital structures [18]. Electrical insulation of the electrode sides where the highest electric field line density occurs, but thermal effects cannot be allowed, might suppress unintended temperature effects and might contribute to a better control where the ablation zone is formed. Areas of high electric field line density also have the highest electric current density, corresponding to the risk of thermal damage [19]. Visual and computational models can be used to provide insight into how the electric field line pattern and temperature gradient can be adjusted with partial electrical insulation of the IRE electrodes. Visualization is the most powerful tool to create understanding of the impact of thermal effects, since IRE could be experienced as a black box by physicians. The aim of this research was to investigate and visualize the change in both the electric field line pattern and temperature gradient to improve the control on the temperature distribution and to identify the potential emergence of new areas at risk for thermal damage, due to the application of partial electrical insulation of the tip and active needle length of the irreversible electroporation electrodes.

## 2. Methods

The effects of electrically insulating specific parts of the IRE electrode were investigated and visualized using the color Schlieren method in a transparent polyacrylamide (PAA) gel, via a semolina in castor oil model, and supported by computational models [16,20].

### 2.1. Electrode Insulation Design

Two monopolar 19 Gauge IRE electrodes (AngioDynamics, Latham, New York, NY, USA) with 1.5 cm active needle length (ANL) were used as reference to investigate the influence of several electrical insulation designs.

**Figure 1:**
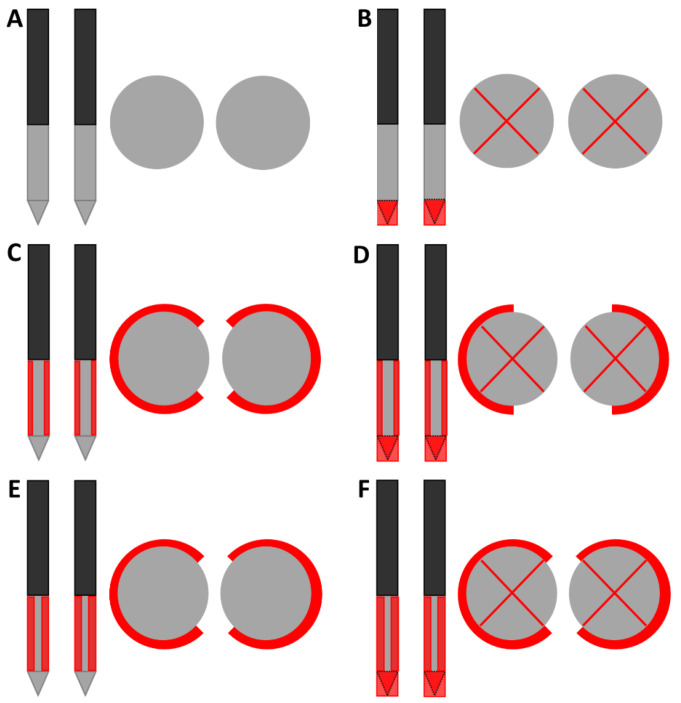
Designs of electrical insulation applied to the IRE electrodes in frontal and transversal view (drawing not to scale). The original IRE electrode (**A**) was used as reference to investigate the effect of electrically insulating the electrode tip (**B**) and half or three-quarters of the circumference of the ANL without (**C**,**E**) and with electrode tip insulation (**D**,**F**). The black, grey and red colors of the schematic overview of the insulation designs represent the original electrical insulation of the ANL, bare needle-electrode area and additionally electrically insulated parts of the ANL, respectively. The insulated tip in the transversal view was imaged as an electrode with a red cross in the middle. In all designs, an ANL and inter-electrode distance of 1.5 cm were used.

Including the original IRE electrode, six designs of electrical insulation were investigated (Figure 1): insulation of the electrode tip (B) and insulation of half or three-quarters of the circumference of the ANL without (C and E) and with partial electrode tip insulation (D and F). In the case of ANL shielding, the part of the ANL that was not facing the other electrode was insulated for half or three-quarters of the needle circumference. Original IRE electrodes were adjusted to create the five insulation designs. Electrical insulation was applied to both electrodes symmetrically. A heat-shrinkable sleeve with glue inside was used to cover the tip when electrical insulation was only applied to this part of the electrode. When the tip was insulated in combination with a part of the ANL, the insulation sleeve of the IRE electrode (kapton) was pushed down in such a way that it completely covered the electrode tip and sealed with polyethyleentereftalaatglycol (PETG), an electrical insulator [21]. The insulation sleeve was incised to create an ANL of 1.5 cm while covering the predetermined circumference of the electrode. In case only the ANL was partially insulated, a small transition zone of 1 mm of insulation sleeve between the unshielded tip and partially insulated ANL was preserved. In this zone, the insulation sleeve was kept intact to prevent the rupture of the insulation material during needle insertion.

### 2.2. Semolina in Castor Oil Model

Semolina is a dipole and will align according to the electric field lines when it is present in a dielectric medium, castor oil, while a static electric field is applied. The method used to visualize the electric field lines is described in a previously published article by our research group [16]. In the current research, a static electric field of 5.4 kV (3.6 kV/cm) in combination with three cameras placed in three orthogonal planes, the frontal, longitudinal and transversal plane, was used to visualize the electric field line pattern around the electrodes for the electrical insulation designs. In all experiments, an inter-electrode distance and active needle length of both 1.5 cm were used, whereby the negative electrode was grounded and the other one was positively charged. Each electrode insulation design was measured once.

An electric field Is present everywhere around the needle–electrode pair when a voltage is applied. The local electric field strength is dependent on the applied voltage, length of the electric field line and local density of field lines [19]. Longer electric field lines were not visible through the semolina in castor oil method since the electric field strength was not strong enough to exceed the gravitational force on long semolina strings and, therefore, more semolina could not be caught. The electric field line density is determined by the number of electric field lines present per area. The more field lines are present, the higher the electric field strength, and the higher the current density could be (in an electrically conductive medium) since electrons flow tangentially along electric field lines.

### 2.3. Polyacrylamide Gel

**Figure 2:**
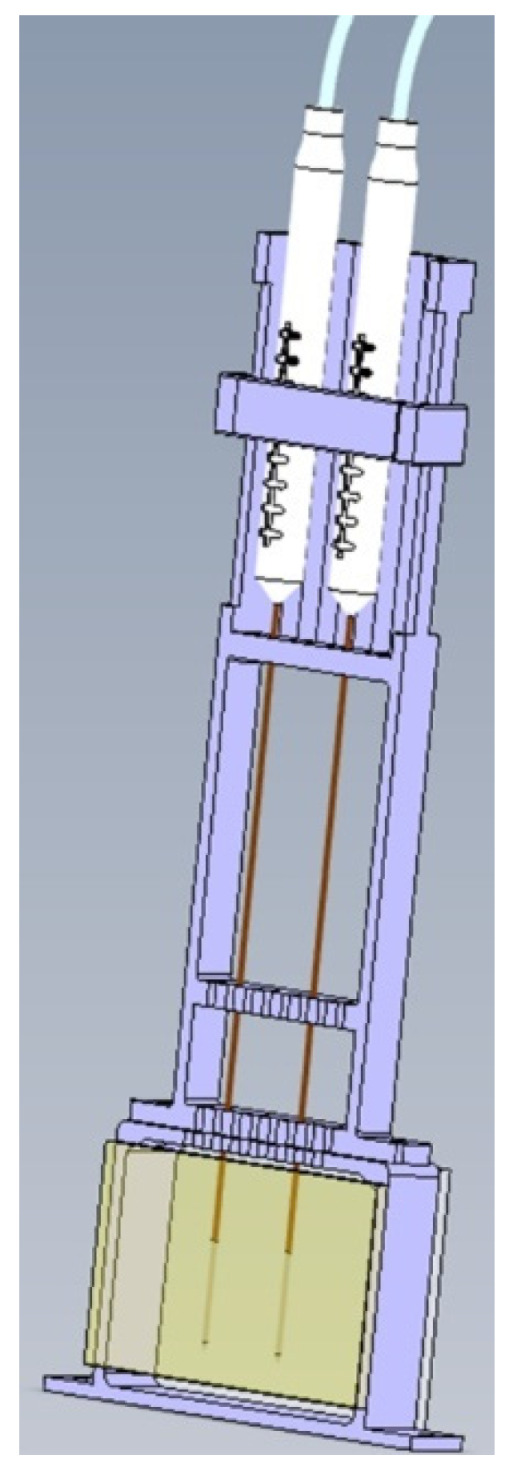
The gel was placed in a holder (purple) to secure the inter-electrode distance. Glass plates (yellow) were used to protect the gel. An inter-electrode distance and active needle length of 1.5 cm were used during the experiments.

A transparent polyacrylamide (PAA) gel was used as a tissue phantom to visualize the temperature gradient during IRE through the color Schlieren method. Polyacrylamide gels have thermal, mechanical and electrical characteristics resembling biological tissue and are, therefore, suitable as a tissue phantom [20]. The ratio of the substances used to produce 100 mL of transparent gel was 60 mL saline solution (NaCl 0.9%), 40 mL 30% acrylamide/bis solution (ratio 37.5:1 (2.7% crosslinker)), 50 mg ammonium persulfate (APS) and 100 µL tetramethylethylenediamine (TEMED). Saline enables the conduction of an electrical current in the gel. TEMED and APS were used as catalysts for the polymerization of acrylamide and bis-acrylamide [20,22]. The substances of the gel were gently mixed and poured in an acrylonitrile butadiene styrene (ABS) mold. Glass plates were placed at the two largest surfaces inside the mold to enable a flat gel surface. The gel was placed in a 3D-printed electrically non-conducting polylactid acid holder (50 × 60 × 15 mm^3^ (length × width × depth)) to secure the inter-electrode distance (Figure 2) [21]. To protect the gel, thin glass plates were placed at the front and back of the gel.

### 2.4. Color Schlieren Method

**Figure 3:**
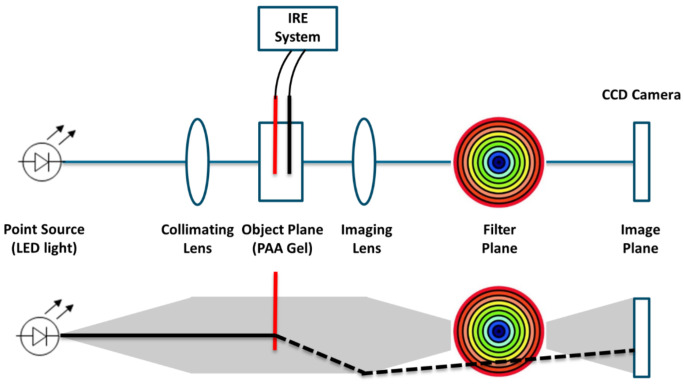
Schematic representation of the color Schlieren setup to investigate the temperature gradient.

The color Schlieren method, a technique to visualize the temperature gradient during IRE pulse delivery, was used to image the temperature development in real time [20]. A change in the temperature of the gel results in a change in optical density and refraction index, which can be visualized via spatial filtering. Figure 3 presents a schematic overview of the color Schlieren setup. An LED was placed at the focal point of a collimator to create a parallel beam of light. Successively, the light rays will pass the optically transparent PAA gel, a focal lens and an optical transparent rainbow filter. This technique relies on the premise that the light rays remain parallel while passing the PAA gel when no electric pulses are delivered, since no change in temperature or refraction index has occurred. In this situation, most of the light will be blocked in the dark center of the rainbow filter. During IRE pulse delivery, the gels’ temperature will increase, resulting in a change in optical density and so the refraction index. The light rays do not remain parallel, refract, will pass one of the outer-colored rings of the rainbow filter and will not be blocked by the center of the filter anymore. The color of the ring through which the light ray passes is depicted in the image plane. This color represents the temperature gradient caused by electric pulsing. The outer rings of the filter (orange/red) visualize a large temperature gradient or large change in temperature and refraction index. The inner rings (blue/green) represent a small temperature gradient.

The BTX Gemini X2 electroporation system (Harvard Apparatus, Cambridge, MA, USA) was used to deliver 20 pulses with 1500 V amplitude and 90 µs pulse length, 10 pulses/s, 1.5 cm inter-electrode distance and 1.5 cm active needle length. The reliability of the Gemini X2 generator to measure the resistance was checked with an 82 Ω electrical resistance, which measured 83 Ω before pulse application and 82 Ω after IRE pulsing. Therefore, this method was considered to be reliable, and the resistance of the medium was measured before and after pulse delivery using the BTX electroporator. The camera (500 frames per second) had a field of view of approximately 15 mm and, therefore, only one of the two electrodes was visualized at a time. Since the change in temperature gradient was visualized around the electrode tip and along the ANL for both the negatively and positively charged electrodes separately, due to the limited camera field of view, the color Schlieren experiment was performed twice per electrode, four times in total per electrode insulation design. Each time, the 20 pulses were delivered to a fresh part of the PAA gel, so that the color visualizations and resistance measurements were not disturbed by the previous pulse delivery.

### 2.5. Image Assessment

The semolina in castor oil and color Schlieren images were visually analyzed and semantically characterized. The electric field line pattern was fully visualized (semolina in castor oil model) thirty minutes after the experiment had started. These images were used for visual image assessment. For the color Schlieren method, the results recorded after the delivery of 5 (highest concentration of electric field lines and highest current density visualized) and 20 (heat diffusion effect visualized) electric pulses were presented in this article. The 20 monopolar IRE pulses were delivered consecutively, and the images after 5 and 20 pulses were extracted from that single recording.

### 2.6. Computational Modelling

**Figure 4:**
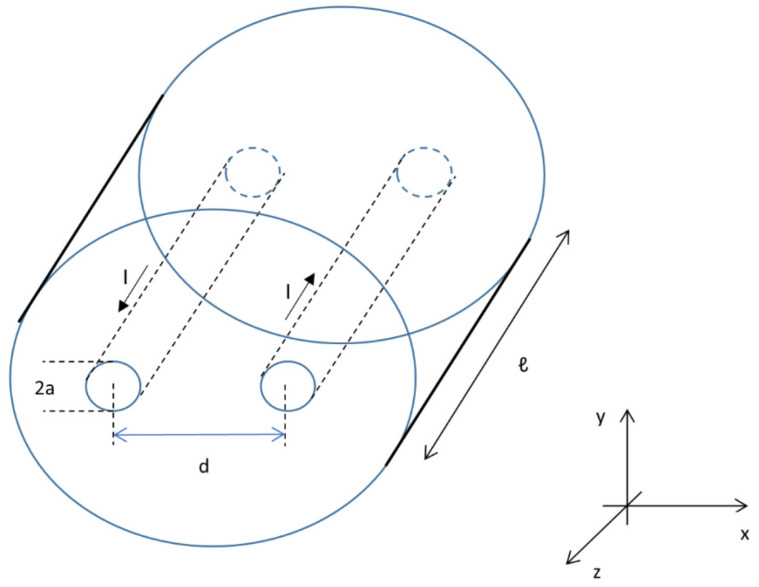
Two needle–electrodes as a conducting cylinder with radius *a*, active needle length ℓ, current flow *I* and inter-electrode distance *d* between the electrodes.

A series of computational models was set up to support and help interpret the experimental results. With this sole purpose, Matlab (Mathworks, Natick, MA, USA) was used for the calculations and visualization of the corresponding results. Figure 4 forms the basis of the computational models used in this research. The needles were considered as two line charges of infinite length with a uniformly distributed charge at their surface since the inter-electrode distance *d >> a*, the electrode radius. Due to this assumption, the proximity effect was absent; coupling of the magnetic fields due to the currents (*I*) inside the needles may be neglected [23]. A cross-section across the needles was suitable to model insulation designs B, D and F (Figure 1). The needle–electrode pair was surrounded by a dielectric medium. A pure capacitor was present for the semolina in castor oil experiments, while a small conductivity was present in the PAA gel that gave rise to a current density field ***J*** in the medium. In both isotropic media, the streamlines or pattern for ***J*** and ***E*** will be the same. According to Ohm’s law, ***J*** = σ***E***.

### 2.7. Resistance

**Figure 5:**
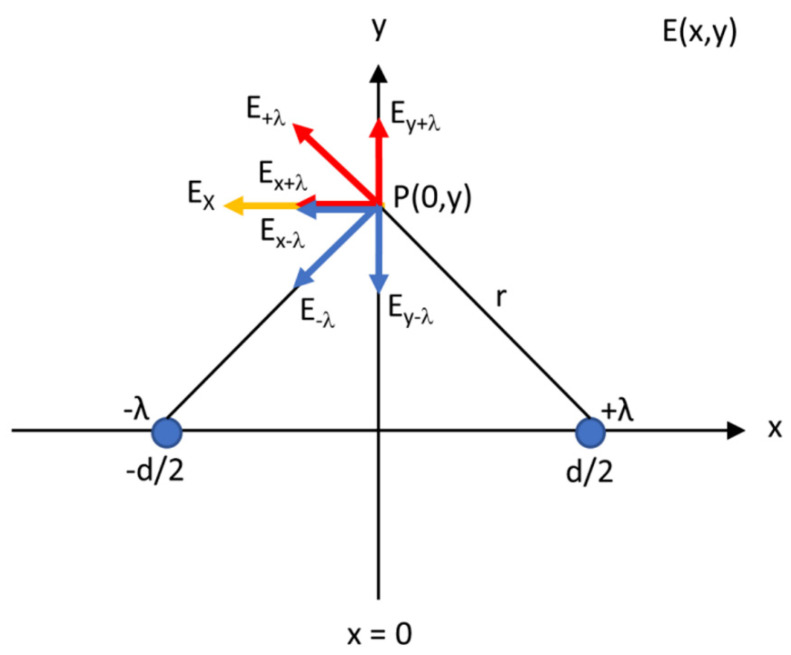
Two line charges (+λ and −λ) at position (−*d*/2, 0) and (*d*/2, 0) in the *x-y* plane, a cross-section along the active needle length of both needle–electrodes, separated by a distance *d*. The resulting field *E*x in a point P at the *y*-axis is the vector sum of the contributions from both line charges.

The active needle length of both needle–electrodes was considered as a segment of an infinitely long straight-line charge. Figure 5 shows a cross-section (*x–y* plane) of two line charges (λ), positively (+) and negatively (−) charged, expressed in coulombs (C)/m. Both charges together represented a cross-section of the active needle pair separated by the inter-electrode distance *d*. The line charges are located at positions (−*d*/2, 0) and (*d*/2, 0). An electric field, indicated by electric field lines, is formed when a difference in voltage is present between the electrodes. Electric field lines are always directed from the positively charged electrode towards the negatively charged one. They are oriented perpendicularly to the electrode surface.

Electric field lines were used to visualize and analyze electric fields as a pictorial tool; it is not a physical entity. The number of electric field lines leaving a positive charge or entering a negative charge was proportional to the magnitude of the charge. Furthermore, the spacing of the electric field lines was inversely proportional to the strength of the electric field. The direction of the field line was tangential to the electric field at any point in space. However, a strict 2D geometry was modelled in this research. The electric field lines were visualized in one plane only and not in 3D. Of course, this is not quite true for a finite active needle length. The *z*-component of the electric field *E*z was quite small for the used configuration of two parallel wires, which constitutes a small capacitor itself.

The electric field in volts (V) per meter in the radial direction (*E*_radial_) perpendicular to the electrode surface is described using Equation (1) [24].

(1) $$E_{radial}=\frac{\lambda }{2\pi \varepsilon_{r}\varepsilon_{0}r}$$

where ε_r_ is the relative permittivity, *ε*_0_ the permittivity of free space in farad (F) per meter (permittivity of the material of interest ε_s_ = ε_r_ε_0_) and *r* the vector distance between one of the line charges (±*d*/2, 0) and point P somewhere on the *y*-axis in the field around the charges.

Equation (2) describes the electric field in the *x-y* plane *E*(*x*,*y*) and in the *x*- and *y* -direction, where $\hat{x}$ and $\hat{y}$ denote the unit vectors in the *x*- and *y*-directions, respectively [24].

(2) $$\begin{array}{l} E\left(x,y\right)=\frac{\lambda }{2\pi \varepsilon_{r}\varepsilon_{0}}\left(\frac{\left(x-\frac{d}{2}\right)\hat{x}+y\hat{y}}{{\left(x-\frac{d}{2}\right)}^{2}+y^{2}}-\frac{\left(x+\frac{d}{2}\right)\hat{x}+y\hat{y}}{{\left(x+\frac{d}{2}\right)}^{2}+y^{2}}\right)\\ =\frac{\lambda }{2\pi \varepsilon_{r}\varepsilon_{0}}\left(\frac{d\left(x^{2}-y^{2}-{\left(\frac{d}{2}\right)}^{2}\right)\hat{x}+2dxy\hat{y}}{\left({\left(x-\frac{d}{2}\right)}^{2}+y^{2}\right)\left({\left(x+\frac{d}{2}\right)}^{2}+y^{2}\right)}\right) \end{array}$$

The electric field on the *y*-axis (*x* = 0) is given by:

$$E\left(0,y\right)=\frac{\lambda }{2\pi \varepsilon_{r}\varepsilon_{0}}\left(\frac{-d\hat{x}}{{\left(\frac{d}{2}\right)}^{2}+y^{2}}\right)$$

and the orientation is in the direction of the negative *x*-axis. The electric field on the *x*-axis (*y* = 0) is as follows:

$$E\left(x,0\right)=\frac{\lambda }{2\pi \varepsilon_{r}\varepsilon_{0}}\left(\frac{d\hat{x}}{x^{2}-{\left(\frac{d}{2}\right)}^{2}}\right)$$

The voltage (*V*) is calculated by integrating the electric field strength between the needle–electrodes, *E*(*x*,0). with ranges −*d*/2 + *a*, a point located on the *x*-axis at the right surface of the negatively charged electrode, and *d*/2 − *a*, a point located at the left side of the positively charged electrode. The voltage is used to calculate the total current *I* in ampere (A) and resistance *R* in ohm meter (Ω m), represented by Equations (3)–(6). Infinitely long line charges (electrodes) and an infinite unbounded PAA gel are assumed for these calculations [19,24,25].

(3) $$V=-\int_{P_{1}}^{P_{2}}E\cdot dl=-\int_{-\frac{d}{2}+a}^{\frac{d}{2}-a}E_{x}\left(x,0\right)dx=\frac{\lambda d}{2\pi \varepsilon_{0}\varepsilon_{r}}\int_{-\frac{d}{2}+a}^{\frac{d}{2}-a}\frac{dx}{x^{2}-{\left(\frac{d}{2}\right)}^{2}}=\frac{\lambda }{\pi \varepsilon_{r}\varepsilon_{0}}ln\left(\frac{d-a}{a}\right)$$

(4) $$I_{total}=\frac{\sigma \lambda }{2\pi \varepsilon_{r}\varepsilon_{0}}\int_{0}^{l}dz\int_{-y_{value}}^{+y_{value}}\frac{d}{{\left(\frac{d}{2}\right)}^{2}+y^{2}}dy=\frac{\sigma \lambda l}{2\pi \varepsilon_{r}\varepsilon_{0}}d\frac{1}{\left(\frac{d}{2}\right)}\tan^{-1}{\left.\frac{y}{\left(\frac{d}{2}\right)}\right]}_{-y_{value}}^{+y_{value}}$$

(5) $$I_{total}=\frac{\sigma \lambda l}{\pi \varepsilon_{0}\varepsilon_{r}}\left(\tan^{-1}\left(\frac{y_{value}}{\frac{d}{2}}\right)-\tan^{-1}\left(\frac{{-y}_{value}}{\frac{d}{2}}\right)\right),\;\mathrm{calculated}\;\mathrm{in}\;\mathrm{radians}$$

(6) $$R=\frac{V}{I_{total}}$$

where *ℓ* is the active needle length, σ the electrical conductivity in siemens/meter, charge λ (coulomb/m) and ***E*** the electric field strength (V/m). Table 1 shows the parameters and corresponding values that were used to calculate the resistance in a computational model. The electrical conductivity of saline, as described by Martinsen and Grimnes (2000), 1.3 S/m, was adjusted by the volume fraction of saline in the PAA gel in Table 1 [26].

The electric field is perpendicular to the *y*-*z* plane in the direction of the *x*-axis (Figure 4). The total current density in that surface was obtained by an integration over the ANL (ℓ) in the *z*-direction and over the furthest point in the *y*-direction (*y*_value_), where the electric field was developed, which differed for the original electrode as well as for half and three-quarters of the circumferentially insulated ANL.

The resistance was calculated before IRE pulse delivery (bare and insulated electrode tip) and after pulsing (insulated electrode tip). The uninsulated electrode tip was modelled by a fictional ANL of 22.5 mm, indicating the space left underneath the electrode tip in the gel to form electric field lines in Figure 2. The electric field was computed at every point in space in the predefined area. The power was defined by:

(7) $$P=J\cdot E=\sigma {\left|E\right|}^{2}$$

Most of the heat is generated near the surface of the electrodes (reference Davalos [28]). The conductivity of the gel increases around 3 percent per degree Celsius. The gel has a low heat transport coefficient (reference Surowiec [29]). The gel close to the electrodes is heated the most when a standard heat model is considered. This model consists of the mass density, specific heat and heat capacity that leads to a temperature change due to the delivered power, leading to the argument that if the conduction increases, more energy will also be absorbed, leading to an increase in temperature. This process was modelled by a larger effective electrode radius of 2.5 mm (*a*_eff_) and decreased distance *d*_λ_ between the line charges, determined via Equation (8), which occurs for each consecutive IRE pulse that was delivered.

(8) $$d_{\lambda }=\sqrt{{\left(\frac{d}{2}\right)}^{2}-a_{eff}^{2}}$$

The resistance (Ω) of the medium was immediately measured by the Gemini X2 generator before and after pulse application during the color Schlieren experiments and compared with the computational value. Both the absolute decrease and the relative percentual decrease in the measured resistance prior to and after the IRE pulse delivery were calculated. Since 4 color Schlieren experiments were performed per electrode design to visualize both the temperature gradient around the electrode tip and ANL of each needle electrode, the resistance prior to and after IRE pulse delivery was measured 4 times as well. The average resistance was calculated and used for interpretation of the results.

### 2.8. Electric Field Line Pattern and Current Pathways

The effect of partial electrical insulation on the electric field line pattern in the 15 mm thick gel slice (bounded medium) and surrounded air (unbounded medium) was visualized for three electrical insulation designs; for the original IRE electrode and needle–electrode pairs, where half and three-quarters of the ANL were insulated. The boundary conditions were continuity of the potential $V_{1}=V_{2}$ and the normal derivative according to $\varepsilon_{1}\frac{V_{1}}{\partial_{n}}=\varepsilon_{2}\frac{V_{2}}{\partial_{n}}$ for a transition from material 1 to material 2. The modelled electric field line pattern was plotted in the transverse plane because that view is least clear to assess in the semolina model due to the distance from the bottom of the tube to the needle–electrode tips.

The gel and needle–electrode pair were placed in a holder of size 50 × 60 × 15 (length × width × depth, all in mm). The size of the holder was indicated on the electric field line pattern to visualize the gel’s boundary.

The field lines or current pathways were plotted according to the free surface charge at the electrodes. The applied voltage of the electrodes was kept constant, so less charge was present at the interface with the insulation. Kapton and the PAA gel have a relative permittivity around 4 and 60, respectively. Therefore, the electrode in contact with the electrical insulation carries a charge that is 15-times lower. The insulation provided a capacitor in series with the gel and the insulation material at the other electrode. The overall capacity will be lower in this case and, therefore, also the charge. The charge distributed over the circumference of the electrode was in proportion to the permittivity at the interface. In addition, there was a fringe effect, which led to an extra, though small, capacity. A field line or current pathway was plotted per 10 degrees at the available bare area of the electrodes. A constant voltage was used in the simulations and, therefore, a constant charge density was present at the bare electrode surface. Of course, there is also an electrical field behind the insulation; however, this field was 15-times smaller than at the bare electrode and did not contribute to the current.

### 2.9. Current Density

The current density was calculated for the original electrode and for half as well as for three-quarters circumferential insulated ANL to identify areas at risk for thermal damage. The electrode tip was not considered in these calculations due to its complex geometry and charge distribution. Equation (9) was used to determine the surface area in mm^2^ of the remaining bare ANL.

(9) S=2πal

With Ohm’s law as the basis, V= *IR*, the total current through the bare surface area of the ANL can be calculated, with the measured resistance prior to IRE pulse delivery (*R*) and difference in voltage between the electrodes (*V*) as input. The current density *j* in ampere/mm^2^ is obtained by:

(10) $$j=\frac{\frac{V}{R}}{S}$$

### 2.10. Temperature

The energy delivery in Joules is dependent on the electric field strength (*V*), induced current (*I*), number of pulses (*N*_p_) [30] and pulse length (*t*_d_) (Equation (11)) [17]. The temperature increase in °C induced by the electric pulses depends on the tissue volume receiving the energy and the energy required to heat 1 cm^3^ of tissue with 1 °C. The characteristics of water were used to calculate the temperature increase in the PAA gel. For water, 1 calorie (4.184 Joule) is required to heat 1 cm^3^ with 1 °C [19]. Equation (12) was used to calculate the temperature increase for the designs with insulated electrode tips. Several assumptions were made to determine the volume where the energy was released. For the electrodes with insulated tips, the volume was bound by the area in between the electrodes and expanded up to 5 mm around the needle–electrode configuration. For insulation of the tip in combination with half or three-quarters (volume was halved) of the ANL circumference, the volume was bound in between the electrodes. The ANL was 15 mm, and the average resistance before and after the delivery of the 20 electric pulses was calculated to determine the average current.

(11) $$Energy=V\times I\times N_{p}\times t_{d}$$

(12) $$∆T=\frac{Energy/4.184}{Volume}$$

### 2.11. Comparison Visualizations and Computational Models

Visualization is the most powerful tool, providing valuable insights for understanding complicated treatments. Both the semolina in castor oil model and color Schlieren technique qualitatively visualized the effect of additional electrical insulation of the IRE electrodes. The electric field line pattern (e.g., fanning out of field lines and density) and color Schlieren pattern (e.g., colors that appeared, area that showed a change in color) were visually interpreted and objectively compared to each other to define the overall effect of the different electrode insulation designs. First-order theoretical models were used as support, to determine if the electrical and thermal effect could be changed by additional electrical insulation, and followed the same trend as observed in the visualizations.

## 3. Results

### 3.1. Semolina in Castor Oil Model

**Figure 6:**
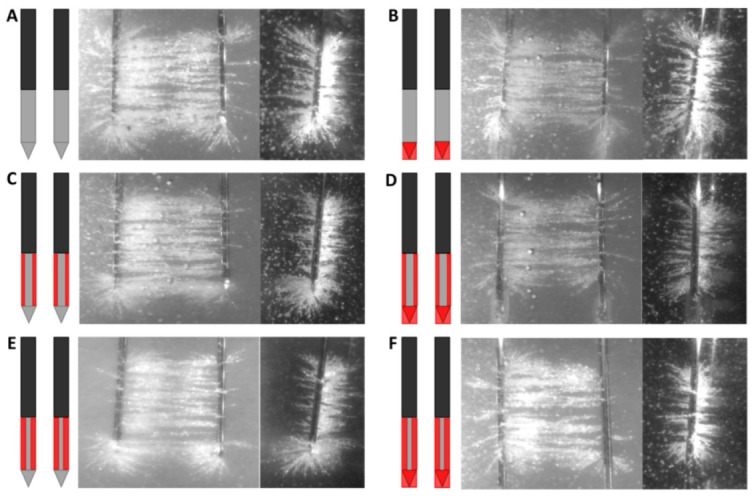
Frontal and longitudinal view of the electric field line pattern visualized by the semolina in castor oil model (**right panel**) for each investigated electrode insulation design: original IRE electrode (**A**), insulated electrode tip (**B**), half or three-quarters of the circumference of the ANL without (**C**,**E**) and with electrode tip insulation (**D**,**F**). The black, grey and red colors of the schematic overview of the insulation designs (**left panel**) represent the original electrical insulation of the ANL, bare needle–electrode area and additionally electrically insulated parts of the ANL, respectively.

**Figure 7:**
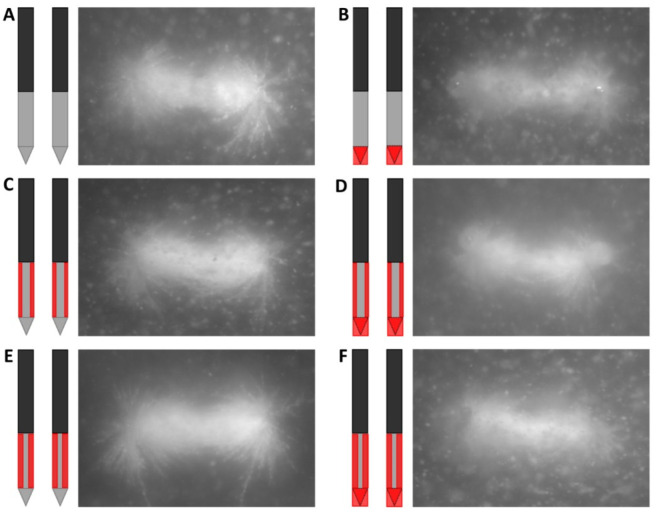
Transversal view of the electric field line pattern visualized by the semolina in castor oil model (**right panel**) for each investigated electrode insulation design: original IRE electrode (**A**), insulated electrode tip (**B**), half or three-quarters of the circumference of the ANL without (**C**,**E**) and with electrode tip insulation (**D**,**F**). The black, grey and red colors of the schematic overview of the insulation designs (**left panel**) represent the original electrical insulation of the ANL, bare needle–electrode area and additionally electrically insulated parts of the ANL, respectively.

The electric field line patterns for the six designs of electrode insulation are shown in Figure 6 (frontal and longitudinal view) and Figure 7 (transversal view). Semolina aligned according to the electric field lines and revealed the electric field line pattern. The original IRE electrode showed a heterogeneous electric field line pattern, formed in three dimensions around the electrodes (Figure 6A). Particularly around the electrode tip electric field lines fanned out in various directions and the highest electric field line density was present in that area (Figure 6A,D,E). This effect was also seen on the transversal images in Figure 7. A more homogeneous electric field line pattern was formed in between the needle–electrodes; electric field lines fanned out less, and a smaller semolina cloud was present around the electrodes for designs where the electrode tip was insulated (Figure 7). The electric field lines were formed around the ANL as well, although fanned out in fewer directions, and presented a lower density in comparison to the field lines visualized around the electrode tip (Figure 6). No electric field lines were visualized around the tip for designs where the electrode tip was insulated (Figure 6B,C,F). Electric field lines were visible at the transition between the electrically insulated part of the electrode and the conductive part of the ANL. No electric field lines became visible at the insulated sides of the electrodes when the circumference of the ANL was partially electrically insulated (Figure 6C–F). When ANL insulation was added, electric field lines were sporadically formed at the transition between the insulation and active part of the electrode in a perpendicular way (Figure 6C,D,F).

### 3.2. Color Schlieren

**Figure 8:**
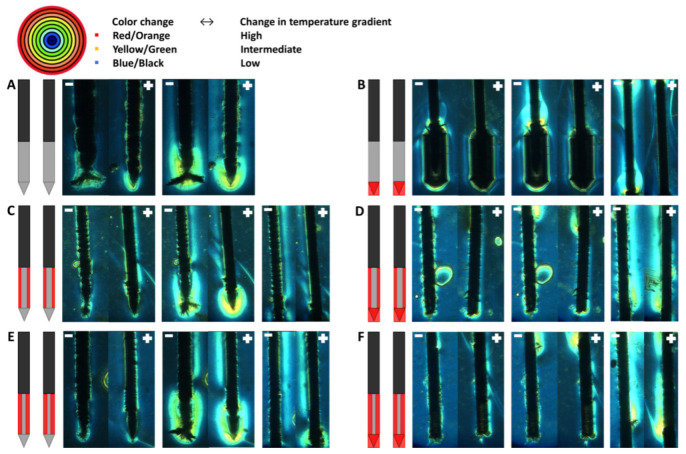
Temperature gradient visualized by the color Schlieren method for every investigated electrode insulation design: original IRE electrode (**A**), insulated electrode tip (**B**), half or three-quarters of the circumference of the ANL without (**C**,**E**) and with electrode tip insulation (**D**,**F**) after the delivery of 5 (**left panel**) and 20 (**middle and right panel**) monopolar electric pulses. The temperature gradient around the ANL after 20 pulses is shown in the right panel. Only a part of the entire ANL could be visualized, the ANL just above the insulated tip or including a part of the active tip, since both the ANL and camera field of view were 1.5 cm. The red outer-most ring of the rainbow filter represents the highest change in temperature gradient, and the black inner-most ring indicates the lowest change in temperature gradient. The black, grey and red colors of the schematic overview of the insulation designs represent the original electrical insulation of the ANL, bare needle–electrode area and additionally electrically insulated parts of the ANL, respectively.

The temperature gradient for the six electrical insulation designs visualized through the color Schlieren method after the delivery of 5 (around the electrode tip) and 20 (around the electrode tip and along the ANL) electric pulses in a PAA gel is shown in Figure 8. Since both the ANL and camera field of view were 1.5 cm, the entire ANL could not be visualized in Figure 8. The temperature gradient first developed at the distal end of the electrode tip after the first electric pulses before expanding to the inner and back sides of the tip, the part of the electrodes not facing each other, and active needle length, respectively.

For designs with uninsulated electrode tips, the first changes in temperature gradient after five electric pulses occurred around the distal end of the tip, with stronger effects around the negatively charged electrode (Figure 8A,C,E). No effects were seen along the ANL of both electrodes. When the tip or both the tip and ANL were partially insulated, the first change in the color Schlieren pattern was observed at the transition between the tip insulation and bare metal of the ANL for the negatively charged electrode (Figure 8B,D,F). Along the rest of the ANL and for the positively charged electrode, no distinct change was visualized. With an increased number of pulses delivered, the color Schlieren pattern expanded and showed higher temperature gradients because of heat conduction.

A symmetric and comparable color pattern was formed for every insulation design after the delivery of all 20 electric pulses. During pulsing, no color change was visualized at locations where electrical insulation was applied (Figure 8B–F). It was observed that the electrode tip caused a stronger change in temperature gradient in comparison to the ANL (Figure 8A,C,E). A higher temperature gradient was observed at the transition area between the electrical insulation and active part of the electrode. This was especially the case for designs where the electrode tip and half or three-quarters of the ANL were insulated (Figure 8B,D,F). In Figure 8B,F, an interruption in the colored area was present in the center of the ANL. A larger colored area with higher temperature gradients was visualized along the ANL when both the electrode tip and ANL were insulated.

For the electrode designs where the tip was uninsulated, the gel ruptured around the negative electrode during pulse delivery. At these locations, the color change was masked. Electric arcing was also observed along the negatively charged electrode, at the electrode tip and at the proximal transition between the electrical insulation material and bare needle-electrode, accompanied by bangs/ticking sounds and light flashes. Most strong effects were seen around the electrode tip of the active tip designs, where the gel was also cracked. The gel cracked after the first few pulses; a small crack was already visible at the color Schlieren images after five IRE pulses.

The more the electrode surface was electrically insulated, the higher the measured resistance before and after pulse delivery (Table 2). Also, the color intensity and colored surface area increased with an increasing amount of electrical insulation applied.

Small inhomogeneities were present during the experiments, including air bubbles between the glass covering and PAA gel, and small spheres or swirls could be visualized in some gels (Figure 8C–E). These inhomogeneities led to local changes in optical density and a through the color Schlieren method visualized change in color.

### 3.3. Mathematical Modelling

#### Electric Field Line Pattern and Current Pathways

**Figure 9:**
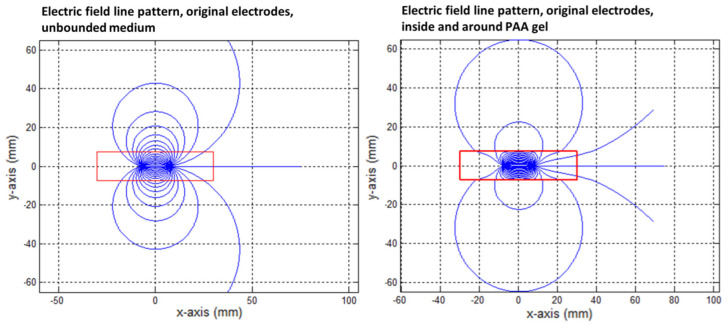
Electric field line pattern for the original IRE electrode in an unbounded medium (**left**) and as formed in the PAA gel slice (**right**). The electrodes were represented by the area showing the highest electric field line density. The red square indicates the size of the PAA gel slice used for the color Schlieren experiments. Electric field lines were plotted per 10 degrees.

**Figure 10:**
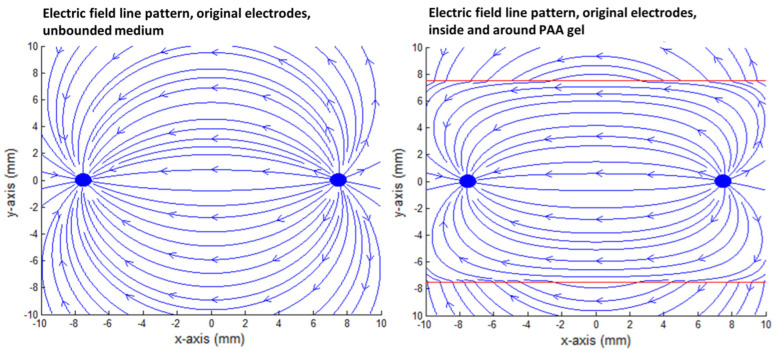
Close-up of the electric field line pattern formed inside the PAA gel slice (**right**) in comparison to the unbounded medium around the gel (**left**) for the original IRE electrode. The red square indicates the size of the PAA gel slice used for the color Schlieren experiments.

**Figure 11:**
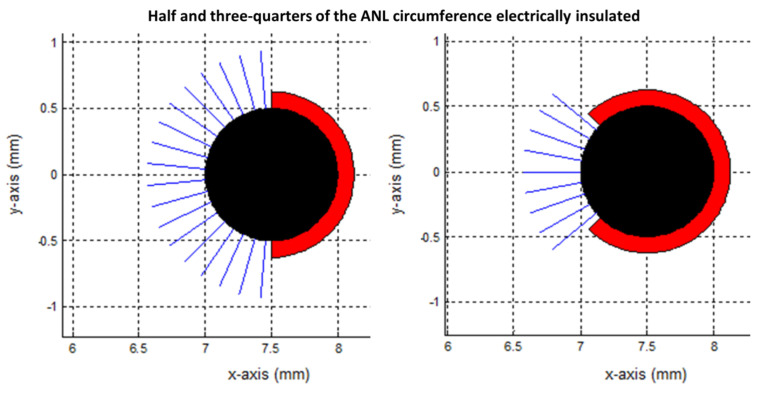
Transversal view of the modelled needle–electrodes where half (**left**) or three-quarters (**right**) of the ANL circumference were electrically insulated. Current pathways leave the electrode surface in a perpendicular way and provide an indication of the current density. Current pathways were plotted per 10 degrees. The blue, black and red colors represent the beginning of the current pathways, bare needle–electrode area and additionally electrically insulated parts of the ANL, respectively.

**Figure 12:**
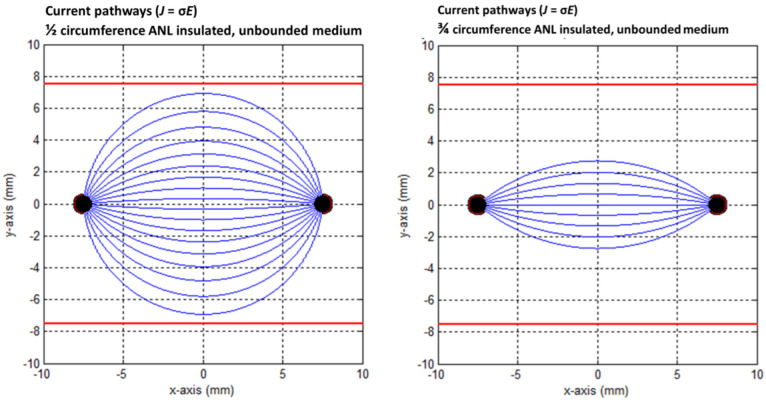
Current pathways when half (**left**) and three-quarters (**right**) of the circumference of the active needle length were electrically insulated in an unbounded medium. The red square indicates the size of the PAA gel slice used for the color Schlieren experiments. Current pathways were plotted per 10 degrees.

**Figure 13:**
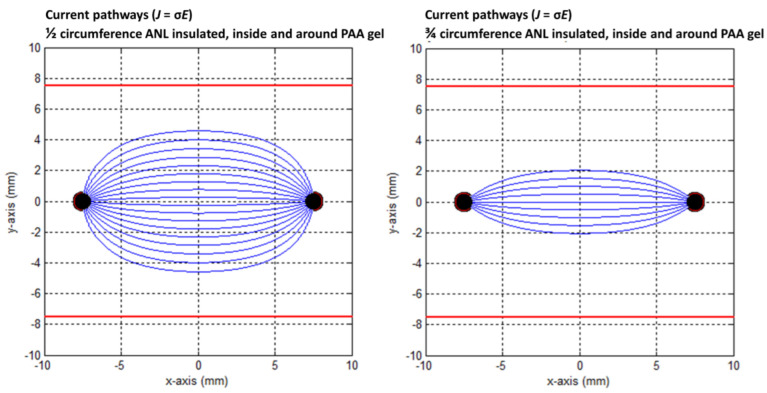
Current pathways when half (**left**) and three-quarters (**right**) of the circumference of the active needle length were electrically insulated in the PAA gel slice. The red square indicates the size of the PAA gel slice used for the color Schlieren experiments. Current pathways were plotted per 10 degrees.

The effect of the PAA gel slice on the electric field line pattern and the current pathways are shown in the transversal direction in Figure 9, Figure 10, Figure 11, Figure 12 and Figure 13 for the original IRE electrodes (*y*_value_ ± ∞) and for the designs where half (*y*_value_ ± 3.129) or three-quarters (*y*_value_ ± 7.5) of the ANL circumference were electrically insulated.

Figure 9 shows that the electric field lines were not only contained to the PAA gel slice, and large-amplitude loops were visible between both electrodes.

In the case of the original IRE electrodes, the long electric field lines from “behind” did not close due to the finite size of the gel. The field lines that were present outside the PAA gel slice did not contribute to the delivery of the electric current between the electrodes. The electric field lines were compressed in the PAA gel and flattened in the corners and at the boundaries of the gel in order to cope with the gel–air boundary condition. This formed a contrast to the medium around the gel, where the electric field lines follow the characteristic loops. Due to the compression, an increased field line density was present inside the PAA gel (Figure 9 and Figure 10).

Figure 11, Figure 12 and Figure 13 show the current pathways in the unbounded medium and PAA gel slice when half and three-quarters of the ANL circumference were electrically insulated. The needle–electrodes were electrically insulated according to Figure 11. Current pathways leave the electrode surface perpendicularly and provide an indication of the local current density.

Electric field lines were present, but no current pathways were formed behind the insulated ANL. The current pathways became more focused when ANL insulation was applied, and only the pathways along the shortest distance between the electrodes were formed (Figure 12). This was supported by the semolina pattern in the longitudinal direction (Figure 6) and transversal direction (Figure 7B,D,F), where a smaller band of semolina was captured around the ANL the more the circumference of the ANL was electrically insulated. Figure 13 shows the compression effect of the PAA gel on the electric field line pattern and current pathways, leading to an increased current density in comparison with the unbounded medium.

### 3.4. Resistance

An overview of the resistance measured before and after IRE pulsing, the absolute decrease and relative percentual decrease in resistance and the computed resistance are presented in Table 2. Both showed the same trend: the more insulation was applied, the higher the resistance prior to IRE pulse delivery. The resistance strongly decreased after pulse delivery. The more electrical insulation was applied, the higher the decrease in resistance. All these effects were visualized within each three pairs of insulation design, e.g., ½ circumference ANL—½ circumference ANL and tip, as well as between the corresponding designs of different pairs, e.g., original IRE electrode—½ circumference ANL—¾ circumference ANL.

### 3.5. Current Density

Table 3 includes the current density for the three electrical insulation designs where the electrode tip was insulated. In contrast to the surface of bare metal, the current density increased when more insulation was applied.

### 3.6. Temperature

Table 4 presents the temperature increase for the electrode designs with insulated tips. The volume where the energy could be released decreased when more insulation was applied to the circumference of the ANL. This resulted in an increase in temperature, unless both the average current and energy delivered into the tissue decreased.

## 4. Discussion

Valuable insights on the effect of partial electrical insulation of the IRE electrodes were provided by the semolina in castor oil model and the color Schlieren method. The visual and, therefore, qualitative experiments were supported by computational models to provide a physical explanation of the observed effects. A heterogeneous electric field line pattern was formed for the original IRE electrode. In the semolina model, a more homogeneous electric field was formed with the increasing amount of insulation applied. The application of partial electrical insulation provided the ability to control where the current pathways were formed during IRE. The electrode tip showed the strongest change in temperature gradient. In line with this finding, the electric field line density and the current density were the highest at the tip. The insulation of the tip strongly decreased the fanning out of both field lines and potential current pathways around the tip and, therefore, could be a solution to control the temperature effect. However, new but potentially less intense hotspots may arise at the transition between tip insulation and bare active needle length. Even a stronger increase in temperature (gradient) was observed at the tip insulation–active electrode transition and along the ANL when insulation was applied to both the tip and ANL. Since the current density and temperature increased with the amount of insulation, it is advised to adjust the IRE parameter settings (e.g., voltage or pulse length) to ensure a safe treatment. Therefore, based on all findings, the electrical insulation of only the electrode tip is a potential innovation to better control the IRE treatment.

New thermal hotspots arose when a larger surface of the electrode was electrically insulated, especially for designs when the electrode tip or both the tip and part of the ANL circumference were insulated. New hotspots were observed through the color Schlieren method at the transition between the electrode tip insulation and bare metal as well as along the ANL. In addition to an increase in the temperature gradient, the modelled temperature increase also rose. Unless the resistance increased and current decreased when more insulation was applied, the local current density and temperature rose as a consequence of the more limited volume between the electrodes.

Our finding contrasts with the work of Yao et al. (2017), wherein a lower temperature rise was measured for the electrodes that were electrically insulated for half of the ANL circumference and outer side of the electrode tip (∆T less than 3 °C) compared to the original needle–electrodes (∆T less than 4 °C) [30]. A fiber-optic sensor placed in the middle between the electrodes was used for the temperature measurement of 90 pulses of 1500 V and 100 µs duration. In contrary, the literature and this study visualized that the highest electric field line density is present near the electrodes (tip), and the highest temperatures are expected at that location [31,32]. This may give an explanation as to why no significant difference in temperature was measured.

It should be noted that new hotspots will arise at the “inner” side of the electrode when insulation is applied to both the ANL and tip. That, especially, is the location where the physician wants to achieve the treatment effect. When insulation is applied to the tip only, an increase in ∆T and temperature gradient will occur at the transition between the tip insulation and bare ANL. The area that showed a change in temperature gradient was enlarged, although the temperature gradient at the transition area was less when compared to the effect seen around the electrode tip for uninsulated tip designs. New hotspots could be acceptable on the condition that the temperature increase does not carbonize the tissue, which is a requirement for the current to flow between the electrodes. Accordingly, the applied voltage or pulse length may need to be adjusted to reduce the current density and overcome these unwanted effects but still exceeding the threshold to irreversibly electroporate the tissue [28,33]. The occurrence of new thermal hotspots is an interesting topic to be investigated in future research.

The electrode tip has a complex geometry and large surface and, therefore, carries most charge in comparison to other parts of the electrodes. Also, the metal at the insulation–bare needle transition carries more charge in comparison to the metal halfway to the ANL due to the expelling character of charges [19]. This could explain the indentation in the color Schlieren pattern halfway to the ANL.

A smaller ablation zone (mm^2^) will be formed when half of the tip and the ANL are electrically insulated in comparison to the original needle–electrodes [30]. The ablation zone is hypothetically bounded by the area in between the electrodes and will expand up to 5 mm around the needle–electrode configuration when the original IRE electrodes are used [34]. Therefore, clinicians should be aware of a potentially smaller ablation zone that expands less around the partially electrically insulated electrodes.

Electric arcing was observed due to the presence of lightning bolts around the negatively charged electrode during IRE pulse delivery. Hydrogen is produced around the cathode, and oxygen is formed around the anode, leading to a small gap between the electrode and PAA gel [35,36]. A wider gap is formed around the cathode since hydrogen occupies a volume that is twice as large as the volume encountered by oxygen [35]. This may result in electric arching and higher temperatures (gradient) around the negatively charged electrode, leading to the ruptures in the PAA gel. This finding corresponded to the presence of semolina visualizing the highest electric field line density around the electrode tip and a more intense semolina cloud around the negatively charged electrode in comparison to the electrode of positive charge.

Both the color Schlieren and semolina in castor oil method are intended as a qualitative visualization method for direct comparison between different settings, which could provide indirect quantitative data. For the color Schlieren method, this was supported by two previous studies investigating the temperature effect of different laser settings for vocal cord treatment. It was concluded that the color Schlieren method in combination with PAA gel was a good predictor of the effect in vocal cords, examined through a comparison with histological results [37,38]. It is possible to derive relative quantitative data, like the extent of a thermal field, duration of thermal relaxation (color Schlieren), area where electric field lines appear and information about the local field line density (semolina). Relative comparison and validation with the modelling might be possible but was beyond the scope of this research. Quantitative data could also be gathered by thermocouples or a thermocamera [39]. Since thermocouples are limited to temperature measurements at one single point around the electrodes and, above all, disturb the color Schlieren visualization, and a thermocamera is limited to the temperature at the surface, these methods were not used to quantify the data.

The main strength of this research is the visual insight that was provided in the IRE procedure in general and influence of different designs of needle–electrode insulation on the electric field line pattern and temperature gradient. This enables the physician to take a step forward in understanding, controlling and personalizing IRE treatment.

A few limitations are present in this study. The computational modelling was simplified and based on assumptions. The needle–electrodes were considered as infinitely long line charges, and the electrode tip was not modelled due to its complex geometry. The current density was calculated based on the resistance prior to IRE pulse application. However, the resistance decreases after pulsing due to heat development and the electroporation effect (during IRE treatment of patients), which will lead to an increase in current and current density, accordingly. The current increase, of course, is also dependent on the change in the medium’s conductivity, which is temperature-dependent. To determine the temperature increase, a homogeneous temperature distribution was assumed in a relatively large volume. However, in practice, most of the energy will be delivered closely to the electrodes, leading to a potentially higher temperature increase. These decisions influenced the results, but due to their resemblance with the semolina in castor oil and color Schlieren results, they still are of importance to help us understand the visual results. All electrical insulation designs were investigated once with the phantom models since these experiments provide qualitative information instead of quantitative results. Although the semolina model was validated and PAA gel has electrical and mechanical characteristics resembling human tissue, the results are not directly comparable to in vivo tissue due to its heterogeneous characteristics. In the interpretation of the results of this study, it is important to consider the characteristics of the PAA gel compared to the characteristics of living tissue. Two examples will follow.

Firstly, a drop in resistance occurred after IRE pulse application for every insulation design, but the cause of this decrease has a different origin between the gel phantom and living tissue. Since a PAA gel is acellular, the decrease in resistance is due to an increase in the conductivity of 0.9% saline, the electrical conducting part of the PAA gel, as a reaction to the observed temperature increase [40,41,42]. Beside the temperature rise, permeabilization of the cell membrane also has an important role in the decreased resistance and increased conductivity in living tissue [41,43].

Secondly, the prerequisites to use the color Schlieren method have consequences. These prerequisites include a transparent, homogeneous and thin slice of gel, required to let the light rays pass and, compared to clinical practice, an adaptation in both the voltage (reduced) and pulse frequency (increased) for IRE [20]. The finite size of the gel had a compressing and flattening effect on the electric field lines, leading to an increased electric field line density, which could lead to larger temperature gradients than actually occur. In vivo and tumor tissue, on the other hand, are heterogeneous instead of homogeneous, demonstrate variation in local electrical and thermal conductivity and blood vessels are present in the surrounding area that enable cooling of the ablation zone and can act as an electric field sink [44]. The parameter settings had to be adapted to keep the energy release under control and prevent immediate rupturing of the gel. It can be comprehensively stated that the color Schlieren method serves as an elegant approach to obtain visual insight into IRE, although it has to be interpreted with caution.

The first steps of the innovation process were taken, but it is important to investigate if this new type of IRE electrode electrical insulation design is feasible for implementation in clinical practice. In terms of optimization the insulation design (which insulation design is clinically relevant and which biocompatible insulation material is suitable to use) and safety (which temperatures are reached in the thermal hotspots that arise due to additional electrical insulation, what value in IRE parameter settings (e.g., voltage) is recommended to safely ablate, what are the implications for electrode placement and what is the effect of partial electrical insulation on the formed ablation zone?). The temperatures reached during IRE and development of the ablation zone size are dependent on the presence and configuration of multiple needle–electrode pairs together. The present research was focused on the effects of additional electrode insulation around the tip and the part of the ANL just above the electrode tip. In future research, it would also be interesting to investigate the induced or changed temperature effects at the proximal transition between the electrical insulation and bare electrode since vital structures could be located along the entire ANL.

## 5. Conclusions

Partial electrical insulation applied to the tip of the original IRE electrodes could be a potential solution to reduce the present influence of the electrode tip and improve the predictability of the current pathway development between the electrodes. Due to the complex geometry of the tip, it carries most of the electrical charge compared to other parts of the needle–electrode. Therefore, the tip showed the highest density of electric field lines and current pathways. In combination with partial electrical insulation, the IRE parameter settings may be adjusted to overcome the rise of new thermal hotspots. Further research on the method of electrically insulating the tip and its clinical applicability will show the future of this innovation.

## Figures and Tables

**Table 1:** Computational model parameters used to calculate the electrical resistance prior to the pulse application, based on the color Schlieren experiments in a polyacrylamide gel phantom.

Parameter Name and Symbol	Value	Unit
Electric charge λ	1	C/mm
Electrical conductivity σ	1.0 (saline 0.9% [26])	S/m
Voltage V	1500	V
Active needle length ℓ	15	mm
Relative permittivity ε_r_	60 [27]	-
Permittivity free space ε_0_	8.854 × 10^−12^ [19]	F/m
Inter-electrode distance *d*	15	mm
Radius needle electrode *a*	0.456	mm

**Table 2:** Measured and computed resistance in ohms (Ω) before and after the application of 20 squared monopolar IRE pulses for all investigated electrical insulation designs. Both the absolute decrease in resistance and the relative percentual decrease are shown.

Insulation	Prior Pulsing Measured Resistance (Ω)	Prior Pulsing Computed Resistance (Ω)	After Pulsing Measured Resistance (Ω)	Absolute and Relative Percentual Decrease in Resistance (Ω)	After pulsing Computed Resistance (Ω)
Original IRE electrode	82	49	67	15 (18%)	-
Insulated tip	125	74	86	39 (32%)	34
½ circumference ANL	96	98	68	28 (29%)	-
½ circumference ANL and tip	166	147	89	77 (46%)	65
¾ circumference ANL	145	195	78	67 (46%)	-
¾ circumference ANL and tip	245	292	111	134 (55%)	128

**Table 3:** Measured resistance, computed total current and current density for 3 electrode insulation designs (1500 V).

Insulation	Measured Resistance (Ω)	Total Current (A)	Electrode Bare Surface (mm^2^)	Current Density (A/mm^2^)
Insulated tip	125	12	15 π	0.25
½ circumference ANL and tip	166	9	15 π × 0.5	0.38
¾ circumference ANL and tip	245	6	15 π × 0.25	0.52

**Table 4:** Temperature increase after 20 monopolar electric pulses per insulation design.

	Average Resistance (Ω)	Average Current (A)	Energy (J)	Volume (cm^3^)	∆T (°C)
Insulated tip	105.5	14	37.8	3.75	2.4
½ circumference ANL and tip	127.5	12	29.7	2.25	3.6
¾ circumference ANL and tip	178	8	21.6	1.125	4.6

## Data Availability

The data that support the findings of this study are available from the corresponding author upon reasonable request.
